# Effects of acupoint-stimulation for the treatment of primary dysmenorrhoea compared with NSAIDs: a systematic review and meta-analysis of 19 RCTs

**DOI:** 10.1186/s12906-017-1924-8

**Published:** 2017-08-31

**Authors:** Yang Xu, Wenli Zhao, Te Li, Huaien Bu, Zhimei Zhao, Ye Zhao, Shilin Song

**Affiliations:** 1 Graduate School, Tianjin University of Traditional Chinese Medicine, Tianjin, 300193 China; 2 Department of Gynecology and Obstetrics, Nankai Hospital, Tianjin Academy of Integrative Medicine, Tianjin, 300100 China; 3 Department of Neurology, Nankai Hospital, Tianjin Academy of Integrative Medicine, Tianjin, 300100 China; 4 Department of Chinese Medicine, Tianjin Hearing Impairment Specialist Hospital, Tianjin, 300150 China; 5 Department of Public Health, School of Chinese Medicine, Tianjin University of Traditional Chinese Medicine, Tianjin, 300193 China; 6 Department of Gynecology and Obstetrics of Chinese Medicine, First Teaching Hospital of Tianjin University of Traditional Chinese Medicine, Tianjin, 300193 China; 7 Department of Chemical Engineering, University of Florida, 1006 Center Drive, Gainesville, FL 32611 USA; 8 Institute for Cell & Tissue Science and Engineering, University of Florida, Gainesville, FL 32611 USA; 9 Laboratory of Anatomy, School of Integrative Medicine, Tianjin University of Traditional Chinese Medicine, No. 88 Yu Quan Road, Nankai District, Tianjin, 300193 China

**Keywords:** Acupoint-stimulation, Primary dysmenorrhoea, Meta-analysis, Systematic review, Non-steroidal anti-inflammatory drugs

## Abstract

**Background:**

Primary dysmenorrhoea (PD), defined as painful menses in women with normal pelvic anatomy, is one of the most common gynaecological syndromes. Acupoint-stimulation could potentially be an effective intervention for PD. Our aim was to determine the effectiveness of acupoint-stimulation compared with Non-Steroidal Anti-Inflammatory Drugs (NASIDs) in the treatment of PD.

**Methods:**

Six databases were searched to December 2014. Sixteen studies involving 1679 PD patients were included. We included randomized controlled trials that compared acupoint-stimulation with NASIDs for the treatment of PD. The main outcomes assessed were clinical effectiveness rate, symptom score, visual analogue score, variation in peripheral blood prostaglandin F2α (PGF2α) and side effects. All analyses were performed using Comprehensive Meta-Analysis statistical software.

**Results:**

(1) The total efficacy was better than control group: odds ratio = 5.57; 95% confidence interval (95% CI) = 3.96, 7.83; *P* < 0.00001; (2) The effect of intervention was positive in relieving the severity of PD symptoms: mean difference (MD) = 2.99; 95%CI = 2.49, 3.49; *P* < 0.00001; (3) No statistical difference existed between two groups in terms of a reduction in the VAS: MD = 1.24; 95%CI = −3.37, 5.85; *P* = 0.60; (4) The effect of intervention on the variation in peripheral blood PGF2α between two groups was positive: MD = 7.55; 95%CI = 4.29,10.82; *P* < 0.00001; (5) The side effects of control groups was more than the acupoint-stimulation group: OR = 0.03; 95%CI =0.00,0.22; *P* = 0.0005.

**Conclusions:**

According to this article, acupoint-stimulation can relieve pain effectively in the treatment of PD and offers advantages in increasing the overall effectiveness.

## Background

Dysmenorrhea is the most common gynecologic complaint among adolescent and young adult females. The prevalence of dysmenorrhoea appears to differ across the world, ranging from 80% in Western Australia [1], to 60% in Canada [2], 48.4% in Mexico [3], and 79.9% in Iran [4]. Over 50% of females of reproductive age have painful menstruation; among them, 10% have severe dysmenorrhoea, whereby their monthly lives’ quality is impaired from 1 to 3 days differently [5]. It starts some hours before menstruation and continues for up to 48–72 h, and takes the form of pains and cramps in the lower abdomen radiating towards the inner side of the thighs [6]. Half of such cases experience systemic symptoms, such as nausea, vomiting, diarrhoea, fatigue, irritability and dizziness [7, 8], which reduce the quality of life. The patients with mild-to-moderate pain can manage their pain without drugs or with a small amount of non-prescription drugs. However, approximately 15% of all women experience severe dysmenorrhoea to a level that affects work or study; such women need drugs to relieve their pain [9]. Dysmenorrhea in adolescents and young adults is usually primary, and is defined as painful menses in women with normal pelvic anatomy [10]. In ~ 10% of females with severe dysmenorrhea symptoms, pelvic abnormalities such as endometriosis or uterine anomalies may be found (secondary dysmenorrhea) [11]. This article mainly discusses primary dysmenorrhoea (PD).

In recent years, there are more and more researches about the pathogenesis of PD. In addition to factors relating to the body’s nerve, genetic and immune systems, and psychological/social factors, the pathogenesis is generally considered to be mainly related to two factors: (1) abnormal uterine contraction, and (2) endocrine and metabolic factors. The state of uterine ischemia and hypoxia causes the uterine muscle to contract, increasing intrauterine tension, and so leading to abdominal pain. Patients with abnormal uterine contractions and the subjective feeling of abdominal colic have been consistently reported over time. Many types of molecular endocrine factors play an important role in the pathogenesis of PD, such as prostaglandins (PGs), oxytocin (OT) and vasopressin (VP), β-EPs, nitric oxide (NO), noradrenaline (NE), endothelins, and magnesium and calcium ions. In particular, prostaglandin F2α (PGF2α), cyclooxygenase (COX) metabolite of arachidonic acid, causes potent vasoconstriction and myometrial contractions, leading to uterine ischemia and pain [12].

Treatment for PD includes a variety of pharmacological and non-pharmacological methods. Common pharmacological interventions include Non-Steroidal Anti-Inflammatory Drugs (NASIDs) and oral contraceptives. NSAIDs are widely used as the first-line therapy for females with dysmenorrhoea [13, 14]. However, there are often adverse events associated with the use of NSAIDs, including stomach ache, diarrhoea, nausea, and liver or kidney damage after discontinuing medication [13]. Therefore, many patients with PD are seeking complementary and alternative techniques such as acupoint-stimulation to treat the symptoms of PD [15], which emphasizes stimulating the acupoint(s) to strengthen the body’s endogenetic regulated function, so as to preventing and treating diseases by regulating the meridian system.

Although previously publications have reported that acupuncture-related treatments are effective for primary dysmenorrhea, the evidence is low convincing due to insufficient methodological quality and small sample size. Given the safety of acupoint-stimulation [16], therefore, the purpose of this systematic review and meta-analysis study is to determine the effectiveness of acupoint-stimulation in treating PD.

## Methods

### Search strategy

We searched six electronic databases that included PubMed, the Cochrane Library, Embase, the Chinese Academic Journals Full-text Database, the Chinese Science and Technology Journal Full-text Database (CNKI), Wanfang Data, and the Chinese Biomedical Literature Database (VIP). The index terms were the following: dysmenorrhoea, menorrhagia, painful menstruation, menstrual, pain, painful menstruation, menstrual pain, menstrual pains, acupuncture, moxibustion, auricular point, ear acupoint (administering persistent/temporary pressure with Cowherb seed/finger force to stimulate pressure points), electroacupuncture, acusector, acupoint application, randomized controlled trials, controlled clinical trials, and random. The above terms in Chinese were adapted and searched in Chinese databases. The studies were published between the first year they were available and December 2014, which of the language is Chinese and English.

### Selection criteria and exclusion criteria

#### Selection criteria

- Research Type
- Research Subjects
- Interventions
- Outcomes (*Clinical effectiveness rate, Symptom score, Visual analogue score, Peripheral blood PGF2α, Side effects*)

#### Research type

Randomized controlled trials (RCTs).

#### Research subjects

Patients with a definite PD diagnosis: PD is defined as painful menses in women with normal pelvic anatomy. An eligible patient is diagnosed based on the PD Clinical Guideline of the Society of Obstetricians and Gynaecologists of Canada.

#### Interventions

Intervention groups – acupoint-stimulation, including acupuncture, moxibustion, ear acupressure, electroacupuncture, acupoint application; Control groups – NSAIDs.

#### Outcomes

(1) Clinical effectiveness rate

It was a dichotomous outcome and the overall effectiveness of acupoint-stimulation therapy as a subjective assessment, which was defined as the proportion of participants who got relieved pain and was based on response evaluation criteria used in the treatment of insomnia with acupoint-stimulation. What’s more, it was reported by trial participants themselves. For example, clinical therapeutic effect criteria were categorized as cure, markedly effective, effective, or ineffective. According to the Guideline for Clinical Trials of New Patent Chinese medicines (GCTNPCM) [17] evaluation standards, which define: Cured: after treatment, the score of symptoms was;restored to 0, abdominal pain and other symptoms disappeared and the dysmenorrhea did not recurred 3 menstrual cycles after treatment; Markedly effective: after treatment, the score of symptoms was decreased to less than 1/2 of the score before treatment, abdominal pain obviously relieved and other symptoms improved and the patient without taking analgesics could insist in work; Effective: after treatment,the score of symptoms decreased to 1/2–3/4 of the score before treatment, abdominal pain relieved and other symptoms improved, and the patient could work after taking analgesics; Ineffective: abdominal pain and other symptoms did not change. The total number of “cure, markedly effective, effective” were used to calculate effective rate.

(2) Symptom score

In accordance with the GCTNPCM, the patients’ symptom scores were recorded before and after treatment [18].

(3) Visual analogue score (VAS)

In the paper, we draw a 10 cm above the horizontal line and horizontal line of the end of 0, indicating no pain; on the other side of 10, said the pain; middle part of said varying degrees of pain. Feel the patient according to uniform mark on the horizontal line, indicating the degree of pain [19].

(4) Peripheral blood PGF2α.

The blood was taken from cubital vein within 24 h in the last menstrual period before treatment and within 24 h in the next menstrual period after treatment for one course, and the plasma PGF2α levels in the two groups were determined with radioimmunoassay.

(5) Side effects

To observe the vital signs before and after treatment and whether there were fainting, stomach ache, diarrhoea, nausea, and liver or kidney damage during the treatment and other adverse events occurred, and recorded.

#### Exclusion criteria

1. Trials where it was unclear whether a randomized trial was being conducted;
2. Trials conducted using combinations of treatments and many medical interventions;
3. Trials in which the data were inadequate and difficult to extract.

### Data extraction and quality assessment

Searches were conducted and the data extracted by two independent researchers. Each trial identified in the search was evaluated for design, eligibility criteria for participants, and outcome measures. Any disagreement between researchers with regard to the eligibility of a trial was resolved by consulting a third researcher. We created a form for data extraction which included: (1) basic information about each trial, including the topic, first author, dateline and journal; (2) basic information about the patients, including the number of cases in each group and the mean age; (3) the study design and intervention; and (4) the outcomes.

The quality of the trials included in this study was assessed by other two researchers according to the Cochrane Handbook for Systematic Reviews of Interventions, Version 5.1.0.

### Statistical analyses

All analyses were performed using Comprehensive Meta-Analysis statistical software, RevMan 5.1.0 (Cochrane Collaboration, Copenhagen, Denmark). Continuous outcome variables were analyzed using a standardized measure; dichotomous variables were compared and the results presented as odds ratios/risk ratios (OR/RR).

To obtain a standard deviation of the change from baseline for the experimental intervention, use (R_1_ = 0.5) [20]:

$$ SD(C)=\sqrt{SD{(B)}^2+ SD{(F)}^2-\left(2\times {R}_1\times SD(B)\times SD\times (F)\right)} $$

*SD(B)* represents the standard deviation before intervention; *SD(F)* represents the standard deviation after intervention.

The research team evaluated homogeneity among the trials via I^2^. If I^2^ was ≥50%, the trials were considered to be heterogeneous, and a random-effect model based on a Mantel-Haenszel (MH) or inverse variance (IV) statistical approach was selected. If I^2^ was <50%, the studies were considered to be homogeneous, and a fixed-effects model based on an MH or IV statistical approach was used. Pooled summary statistics of the differences in the ratio or mean of the individual studies were developed. Pooled differences in ratios or means, and two-sided *P*-values were calculated and used as criteria for determining the level of statistical significance. *P* < 0.05 was considered to indicate statistical significance. Moreover, a sensitivity analysis was conducted based on the leave-one-out cross-validation procedure [21].

## Results

### Study selection

**Fig. 1:**
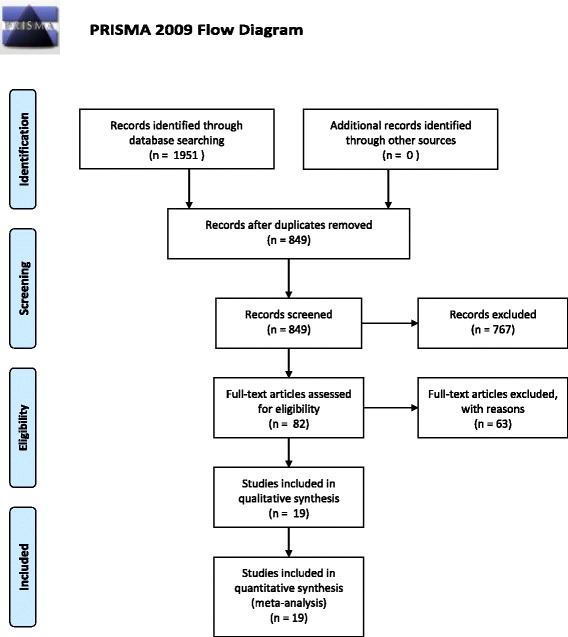
PRISMA 2009 Flow Diagram

A flow chart of the included/excluded studies is shown in Fig. 1. Database searches yielded 70 studies from PubMed, 28 from the Cochrane Central Register of Clinical Trials, 215 from Embase, 552 from CNKI, 328 from Wanfang Data, 279 from VIP, and 479 from CBM. After removal of duplicate records, 849 records remained. Following the first review based on the title, 149 records were remained, and the abstracts were reviewed based on the pre-defined eligibility criteria. A total of 82 records were selected for full text review and data processing. During this phase, 63 papers were excluded, so 19 studies were included in the final meta-analysis, comprising 1679 participants.

### Characteristics of the included studies

Table 1 shows the main characteristics of the 19 RCTs [18, 19, 22–38].

**Table 1:** Characteristics of the 19 Trials Identified in the Literature Search

Studies	Randomization Method	Sample Size Intervention/Control	Age(I/C)	Intervention		Outcomes		Time of initiation of acupoint-stimulation and Course of Treatment	Follow-up Visit
				Intervention group	Control group	Primary	Secondary		
Zhang LM et al. (2012) [22]	Random number table	45/45	13–27/11–25	Acupuncture at SP 10 SP 6 CV 6 LI 4	Indometacin	Clinical efficacy	N/A	The treatment started 3 days before menstrual onset, once every day and was given for 3 days for 3 menstrual cycles	3 months
Lin Q et al. (2012) [23]	Random number table	80/60	15–30/15–30	Eye acupuncture at the lower-*jiao* area; liver area; kidney area; the liver area; the heart area; the spleen area	Ibuprofen	Clinical efficacy	Uterine artery blood flow signals	The treatment started 5 days before menstrual onset, once every day and was given for 4–5 days for 3 menstrual cycles	3 months
Hu YL et al. (2012) [18]	Random number table	60/50	15–30/15–29	Eye acupuncture at the lower-*jiao* area; liver area; kidney area; the liver area; the heart area; the spleen area	Ibuprofen	Clinical efficacy	PGF_2α_+ recurrence rate	The treatment started 2 days before menstrual onset, once every day and was given for 4–5 days for 3 menstrual cycles	6 months
Cao Y et al. (2011) [24]	Random number table	29/30	15–29/20–28	Acupuncture at EX-B8 SP 8 BL 32	Ibuprofen	Clinical efficacy	symptom score+ VAS + side effects	The treatment started during the menstrual period, once every day and was given for 3 menstrual cycles	3 months
Zhi LX et al. (2007) [25]	SPSS Random number	60/60	19.60 ± 3.20/18.93 ± 2.60	Superficial needling at SP 6	Indometacin	Clinical efficacy	symptom score+ analgesic time	The treatment started 3 days before menstrual onset, once every day and was given for 5 days for 3 menstrual cycles	3 months
Bo LN et al. (2013) [26]	Random number table	69/64	13–35	Moxibustion at CV 4 CV 8 SP 6	Fenbid	N/A	VAS + COX + PGF_2α_ + OT + side effects	The treatment started 7 days before menstrual onset, once every day and was given for 7 days for 3 menstrual cycles	3 months
Ren XL et al. (2013) [27]	Registration order	40/40	16–28/18–27	Moxibustion at CV 4 SP 6	Ibuprofen	Clinical efficacy	PGF_2α_	The treatment started 3 days before menstrual onset, once every day and was given for 6 days for 3 menstrual cycles	3 months
Zhu Y et al. (2010) [28]	Random number table	51/51	18–26/19–25	Sandwiched moxibustion at CV 8	Indometacin	Clinical efficacy	symptom score +side effects	The treatment started 3 days before menstrual onset, once every day and was given for 5 days for 3 menstrual cycles	3 months
Li JM et al. (2012) [29]	Random number table	30/30	19–30	Electroacupuncture at BL 32	Fenbid	Clinical efficacy	symptom score	The treatment started during the menstrual period, once every day and was given for 3 menstrual cycles	3 months
Wang K et al. (2005) [30]	Random number table	30/28	16–28/15–24	Ear acupoint at TF 2 CO 18 CO 10 CO 12	Indometacin	Clinical efficacy	N/A	The treatment started 3 days before menstrual onset, once every day and was given for 6 days for 3 menstrual cycles	3 months
Yang M et al. (2009) [31]	Random number table	36/36	14–28/13–27	Acupoint application at CV 4	Indometacin	Clinical efficacy	N/A	The treatment started 2 days before menstrual onset, once every day and was given for 4 days for 6 menstrual cycles	6 months
Chen LW et al. (2006) [32]	Random number table	30/28	16–28/15–24	Acupoint application at CV 4 CV 3 CV 6	Indometacin	Clinical efficacy	N/A	The treatment started 7 days before menstrual onset, once every day and was given for 10 days for 3 menstrual cycles	N/A
Liu C et al. (2011) [33]	Random number table	40/40	21.22 ± 5.86/20.96 ± 6.12	Moxibustion at CV 4 EX-B8	Fenbid	Clinical efficacy	symptom score	The treatment started 7 days before menstrual onset, once every day and was given for 10 days for 3 menstrual cycles	3 months
Zhu C et al. (2011) [34]	Random number table	20/20	17–28/18–27	Acupuncture at CV 4 CV 3 SP 10 SP 8 LI 4 LI 11	Indometacin	Clinical efficacy	N/A	The treatment started 7 days before menstrual onset, once every day and was given for 7 days for 3 menstrual cycles	3 months
Li ZL et al. (2012) [35]	Random number table	100/100	13–30/14–35	Acupoint application at CV 3 CV 8 BL 32 SP 6	Ibuprofen	Clinical efficacy	symptom score	The treatment started 7 days before menstrual onset, once every day and was given for 9 days for 6 menstrual cycles	6 months
Gurkan K et al. (2013) [19]	Registration order	11/24	13.1 ± 1.0/12.8 ± 0.9	Acupuncture at HT 7 PC 6 LI 4 LI 10 SP 6 LR 3 ST 36 GB 26 SP 15	Naproxen sodium	N/A	VAS	The treatment was given three times on the 5th and 2nd days prior to the expected menstruation date and on the third day of menstruation for 1 month	N/A
Jiang LY (2007) [36]	Registration order	34/34	19.35 ± 4.33/20.55 ± 4.51	Acupuncture at BL31 BL32 BL33 LI 3 SP 6 SP 8 CV 4 ST 36	Indometacin	Clinical efficacy	N/A	The treatment started 4 days before menstrual onset, once every day and was given for 7 days for 3 menstrual cycles	N/A
Xing QX (2011) [37]	Registration order	60/54	15–27/16–32	Pricking bloodletting at the liver area;kidney area; the liver area;the uterus area; HT 7	Indometacin	Clinical efficacy	N/A	The treatment started during the menstrual period, once every day and was given for 3 menstrual cycles	3 months
Ji L et al. (2012) [38]	Random number table	30/30	22 ± 3/22 ± 2	Sandwiched moxibustion at CV 8	Indometacin	Clinical efficacy	symptom score+ PGF_2α_ + PGE_2_	The treatment started 3 days before menstrual onset, once every day and was given for 6 days for 3 menstrual cycles	3 months

The above literatures didn't mention intention-to-treat or per-protocol analysis

### Clinical outcomes

#### Clinical effectiveness rate

**Fig. 2:**
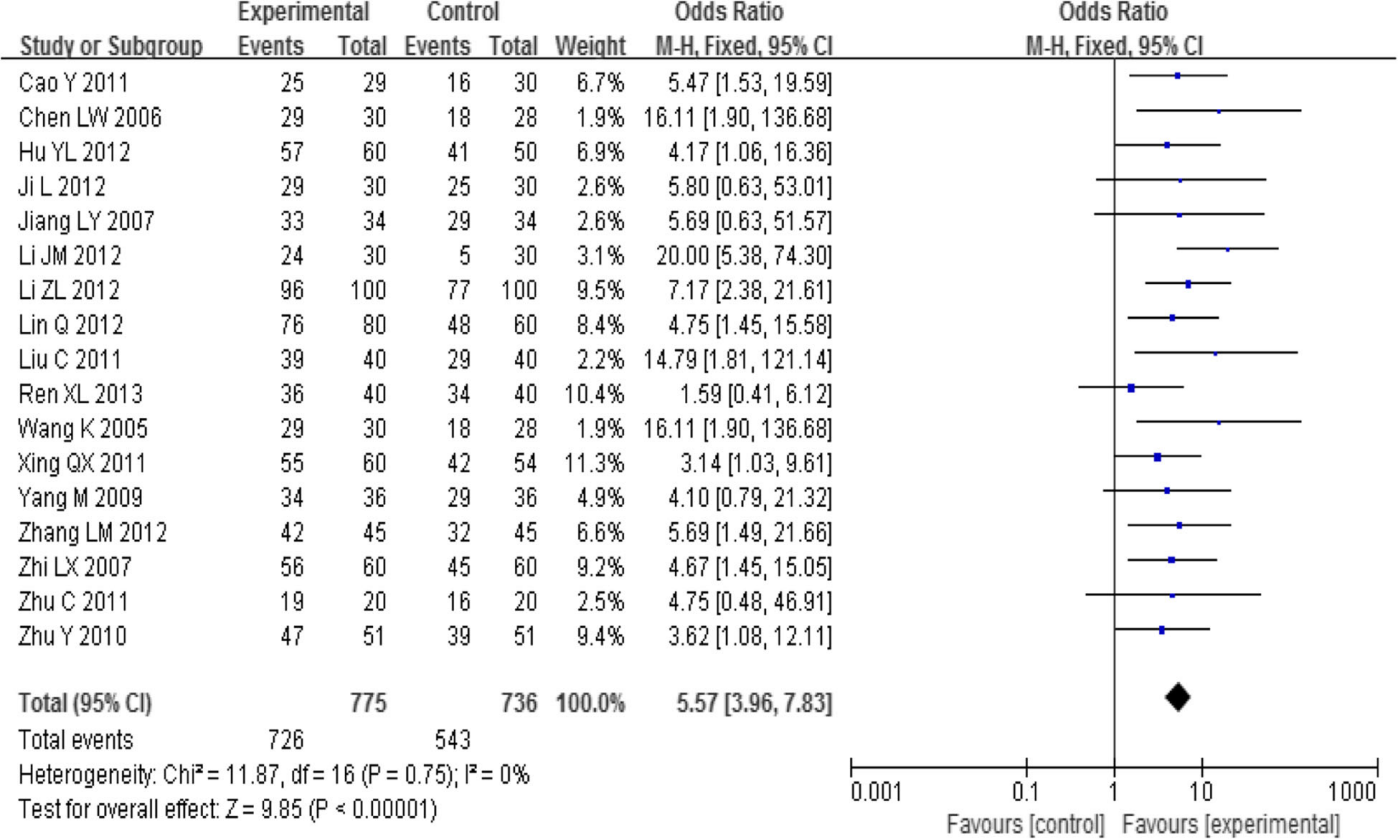
Meta-analysis of the Clinical Effective Rate

Seventeen trials examined the effects of acupoint-stimulation and reported the clinical effectiveness rate of treatment for participants with PD who used those therapies versus the rate for a control group. Analysis of pooled data using a fixed-effect model showed that the effect of intervention on the clinical effectiveness rate was positive [OR = 5.57, 95%CI (3.96, 7.83), *P* < 0.00001] (Fig. 2). That is to say, the clinical effectiveness rate, the acupoint-stimulation group being superior to the NSAIDs.

#### Symptom score

**Fig. 3:**
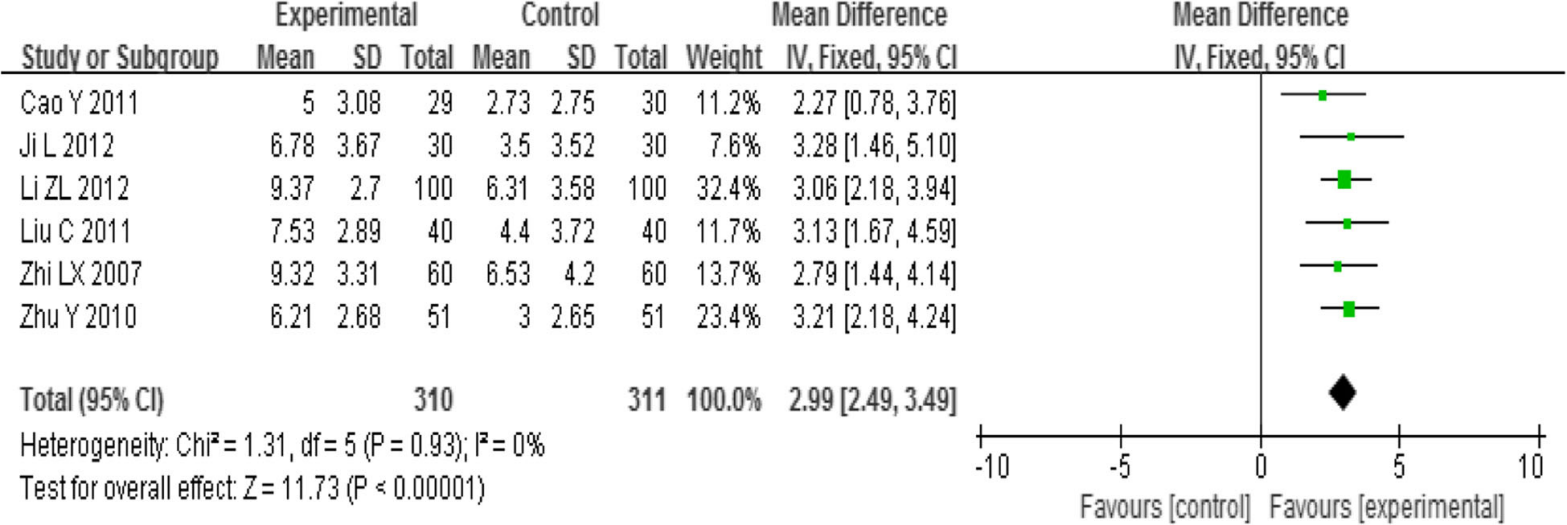
Meta-analysis of the Symptom Score

Six trials reported the symptom score. Analysis of pooled data using a fixed-effect model showed that the effect of intervention on the symptom score was positive [MD = 2.99, 95%CI (2.49, 3.49), *P* < 0.00001] (Fig. 3). The curative effect of acupoint-stimulation on PD is significant.

#### visual analogue score

**Fig. 4:**
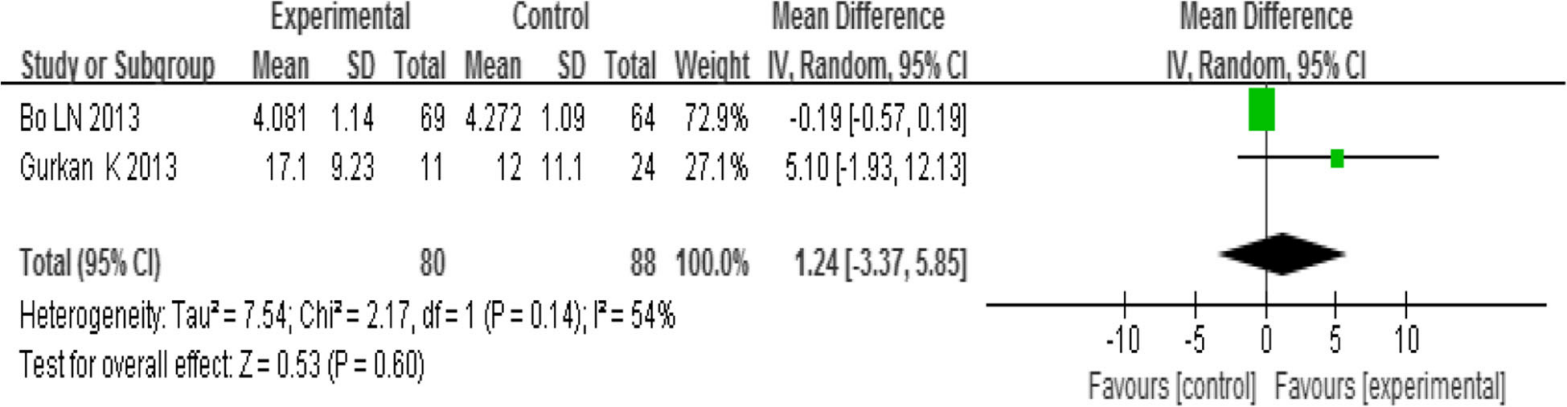
Meta-analysis of the VAS

Three trials reported the VAS; analysis of pooled data using a random-effect model showed that I^2^ = 98%, indicating heterogeneity. So, the trial by Cao (2011) was excluded from analysis, then analysis of the pooled data using a random-effect model showed that there was no statistical difference in variation of VAS between the groups receiving acupoint-stimulation and the control groups [MD = 1.24, 95%CI (−3.37,5.85), *P* = 0.60] (Fig. 4).

#### Peripheral blood PGF2α

**Fig. 5:**
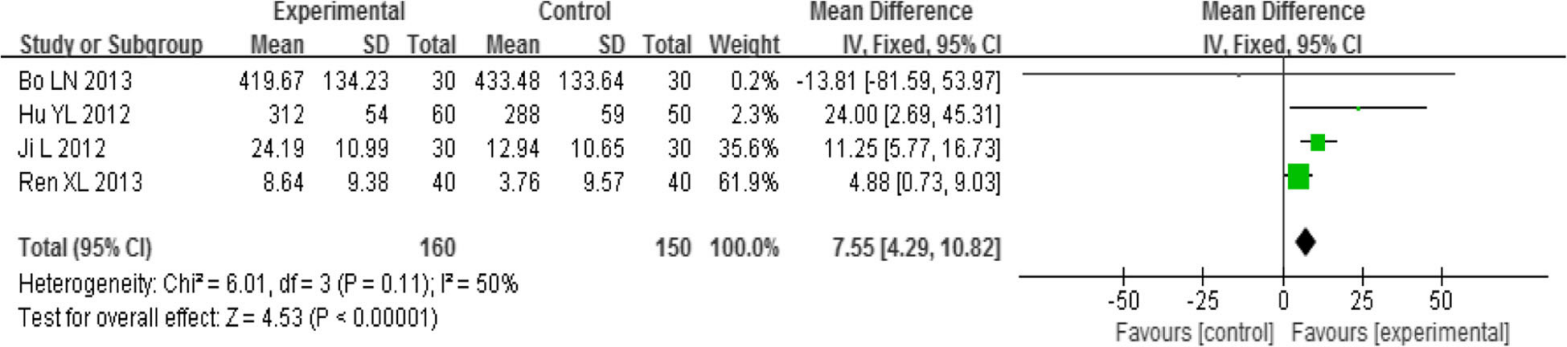
Meta-analysis of the Peripheral Blood PGF2α

Four trials examined the effects of acupoint-stimulation and reported peripheral blood PGF2α of participants with PD who used those therapies versus the rate for a control group. Analysis of the pooled data using a fixed-effect model showed that the effect of intervention on the variation in peripheral blood PGF2α between the groups receiving acupoint-stimulation and the control groups was positive [MD = 7.55, 95%CI (4.29, 10.82), *P* < 0.00001] (Fig. 5). In the study, it is indicated that acupoint-stimulation can effectively decrease peripheral blood PGF2α level in the patient of PD, so as to inhibit PGF2α-induced spastic contraction of uterine muscle, improve the decrease of blood flow, and relieve the symptoms of the patient of dysmenorrhea.

#### Side effects

**Fig. 6:**
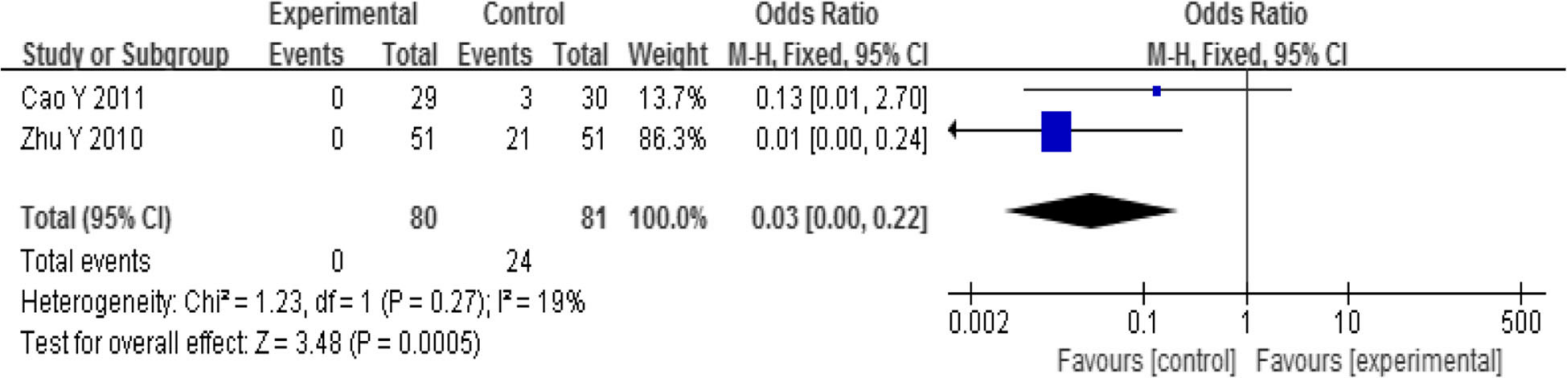
Meta-analysis of the Side Effects

Three trials reported the side effects between acupoint-stimulation and control group. Analysis of pooled data using a random-effect model showed that I^2^ = 83%, indicating heterogeneity. So the trial by Bo (2013) was excluded from analysis, then analysis of the pooled data using a fixed-effect model showed that the side effects of control groups were more than the acupoint-stimulation group. [OR = 0.03, 95%CI (0.00, 0.22), *P* = 0.0005] (Fig. 6).

#### Quality assessment

**Fig. 7:**
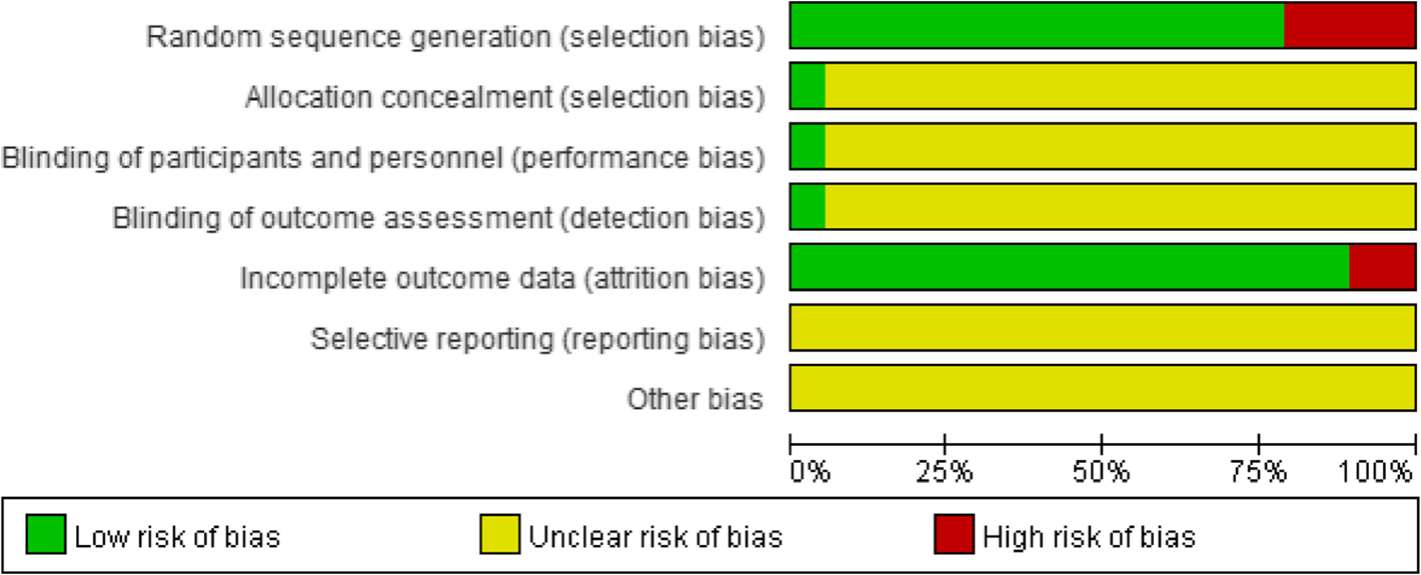
Risk of bias graph: review authors’ judgements about each risk of bias item presented as percentages across all included studies

**Fig. 8:**
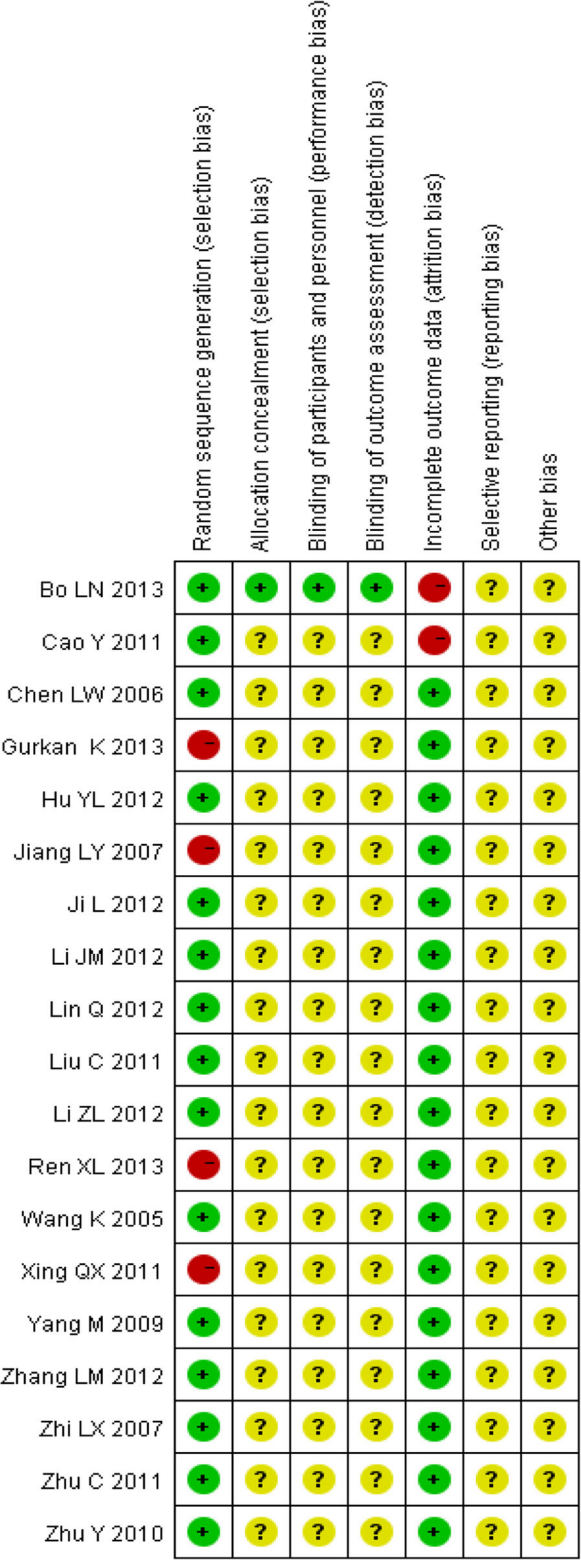
Risk of bias summary: review authors’ judgements about each risk of bias item for each included study

The risks of seven biases among the 19 trials were evaluated, including random sequence generation, allocation concealment, blinding of participants and personnel, blinding of outcome assessment, incomplete outcome data, selective reporting, and other biases according to the criteria in the Cochrane Handbook for Systematic Reviews. Fifteen of the studies described correct randomization methods. There was only one trial with allocation concealment and blinding of participants and personnel and blinding of outcome assessment, and nearly all of the trials failed to mention allocation concealment, the blinding of the participants and personnel, and the blinding of outcome assessments. The methodological qualities of the included trials are summarized in Figs. 7 and 8.

#### Funnel plot of publication bias

**Fig. 9:**
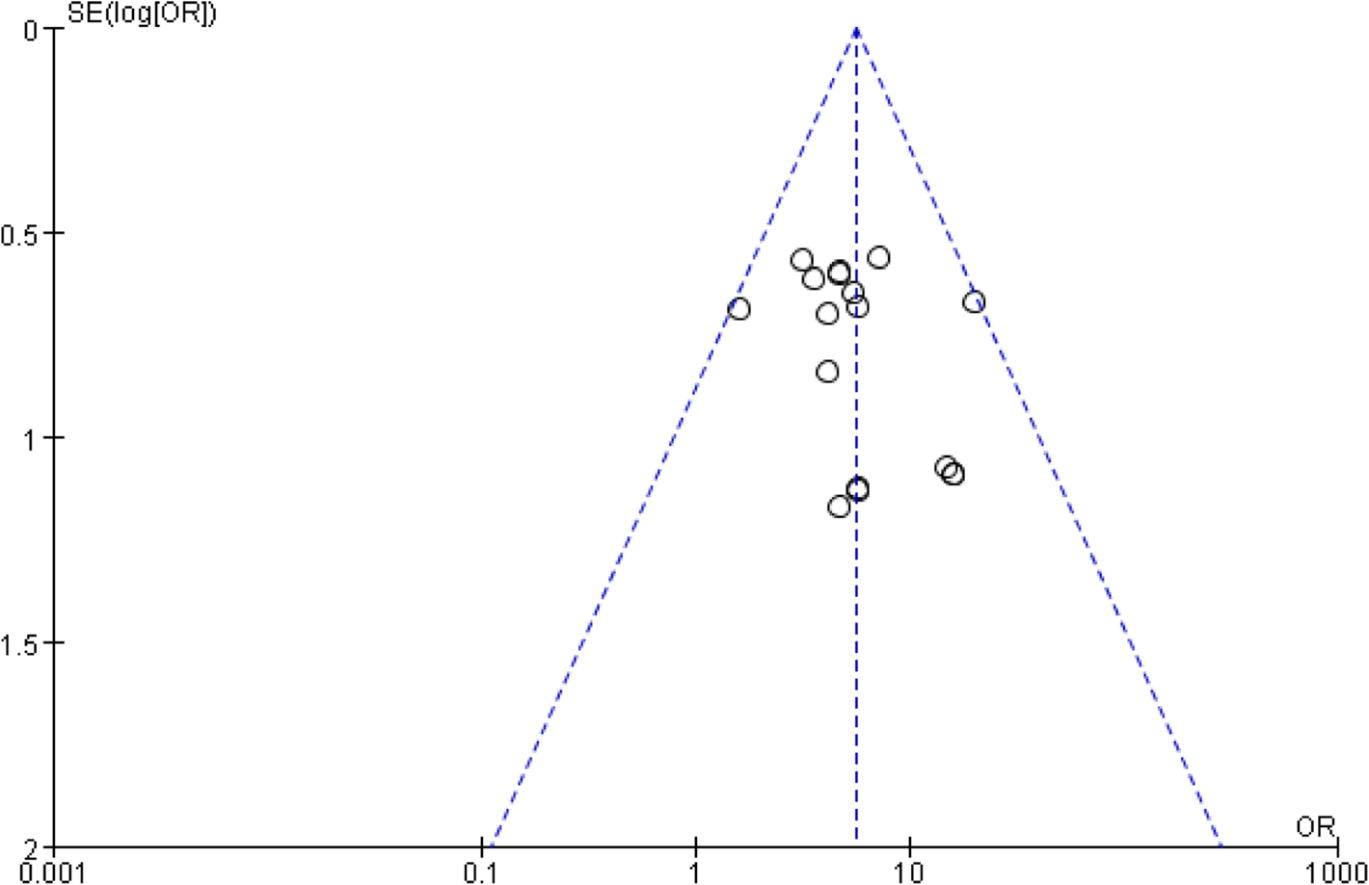
Total Effects of Intervention Groups

The research team performed an analysis of all the included studies, using a funnel plot to determine publication bias in all of the literature. The outcome from the funnel plot analysis is summarized in Fig. 9. The outcome suggests that there was little publication bias.

## Discussion

### Meta-analysis of clinical effect

In the 19 RCTs included, 17 reported a clinical effectiveness rate and 6 reported symptom scores and 4 reported variation in the level of PGF2α in the peripheral blood of women with PD. The meta-analysis revealed that acupoint-stimulation is superior to NSAIDs in the treatment of PD in terms of clinical effectiveness rate and symptom improvement and reducing the concentration of PGF2α in peripheral blood.

Only one RCT reported uterine artery blood flow signals. The results showed that the uterine arterial pulsation index (PI) and uterine arterial resistance index (RI) of the dysmenorrheal patients were significantly increased in the eye acupuncture group before treatment. Moreover, most studies used a subjective, self-reported index of treatment effects as the outcome measure. Because participants self-reported without additional objective outcomes, their pain status could not be assessed accurately [39]. Furthermore, the included studies used different treatments for the intervention group, such as acupuncture, moxibustion, ear acupressure, electroacupuncture and acupoint application, which of the purpose is to highlight the specificity of acupoints.

### The reason for NSAIDs being a drug of positive control

NSAIDs act by inhibiting the enzyme that catalyzes the conversion of arachidonic acid to cyclic endoperoxides, namely COX, which in turn inhibits the production of PGs [40, 41]. The resulting lower levels of PGs lead to less vigorous contractions of the uterus, and therefore to less discomfort. Thus, NSAIDs alleviate primary dysmenorrheic pain predominantly through the suppression of endometrial PGs synthesis [42]. Although NSAIDs is the first-line treatment for PD, it also has shortcomings, which can inhibit the synthesis of COX-1, as well as COX-2, finally it is easy to cause adverse reactions of gastrointestinal and central nervous system. Vane [43] indicated in 1994 that the effective treatment effect of NSAIDs was due to inhibition of COX-2, however, the adverse reactions imputed the suppression of COX-1. Therefore, we consider that NSAIDs may be used as a drug of positive control.

Although the results are encouraging, the conclusions from the current study should be carefully considered before being applied to clinical practice specific patients especially individuals with NSADIs contraindication. This study aims to collect all RCTs relating to acupoint-stimulation treatment of PD and use systematic review to gauge the effectiveness of acupoint-stimulation in the treatment of PD in order to use this treatment more widely in clinical practice.

### Different conclusions of the published literature

Some evidence indicates that acupoint-stimulation is effective in treating primary dysmenorrhea [44–49], but that evidence was largely based on one small, randomized, controlled trial. However, two more recent sham acupuncture randomized controlled trials failed to show evidence of pain reduction [50, 51]. One of the major challenges may be the subjective nature of the symptoms’ presentations and acupoints utilized. Although a few reviews [15, 21, 52, 53] of acupuncture for the treatment of PD are currently available, none of those reviews analyze the potential mechanism of acupuncture for the treatment of PD, which is the key research content in future. Therefore, a systematic review with a meta-analysis is necessary so that quality evidence can be put forward for the use (or not) of acupoint-stimulation for the treatment in individuals with PD.

## Limitations and strengths

The limitations of this evaluation system are as follows: (1) most of the researches did not mention how the sample size was estimated, and most sample sizes were small, leading to a low inspection efficiency; (2) in some of the studies there was inadequate reporting of allocation concealment; implementing or not fully implementing allocation concealment will lead to an exaggerated curative effect; (3) the results were heterogeneous on account of their use of subjective indicators to evaluate the curative effect (symptom scores, VAS), so that implementation of the blinding method is important, but the included studies did not describe the implementation of the blinding method; (4) the study was limited to Chinese and English research, leading to the possibility of selection bias, and the terminology or the guidelines used in clinical managements might not be in the same language.

The strengths of this evaluation system are as follows: this is the first report that comparing the effect of acupoint-stimulation and NSADIs in the treatment of PD, and it provides new evidence and open new horizons that acupoint-stimulation can relieve pain effectively in the treatment of PD and offers advantages in increasing the overall effectiveness.

## Perspectives

In our future research, we will conduct some trials relating to acupoint-stimulation for the treatment of PD, which will focus on the following aspects to prevent bias: (1) an estimation of sample size, (2) a fully random design incorporating allocation concealment, and (3) a blind design for the proposer, performer and measurer.

## Conclusion

The current evidence reveals that acupoint-stimulation in the treatment of PD has some obvious advantages compared with treatment by NSADIs. The advantages are that acupoint-stimulation can alleviate the symptoms of dysmenorrhoea, reduce the level of peripheral blood PGF2α and has fewer side effect, so it can be used to treat PD patients, especially individuals with NSADIs contraindication.

## Abbreviations

CI: Confidence interval
CNKI: Chinese Science and Technology Journal Full-text Database
COX: Cyclooxygenase
COX-2: Cyclooxygenase 2
GCTNPCM: The Guideline for Clinical Trials of New Patent Chinese Medicines
IV: Inverse variance
MD: Mean difference
MH: Mantel-Haenszel
NE: Noradrenaline
NSAIDs: Non-Steroidal Anti-Inflammatory Drugs
OR: Odds ratios
OT: Oxytocin
PD: Primary dysmenorrhoea
PGF2α: Prostaglandin F2α
PGs: Prostaglandins
PI: Pulsation index
PRC: People’s Republic of China
RCTs: Randomized controlled trials
RI: Resistance index
RR: Risk ratios
TCM: Traditional Chinese Medicine
VAS: Visual analogue score
VIP: Chinese Biomedical Literature Database
VP: Vasopressin
